# Myositis Ossificans of the Temporalis Muscle Following Neurosurgical Intervention: A Report of a Rare Case and Literature Review

**DOI:** 10.7759/cureus.87512

**Published:** 2025-07-08

**Authors:** Asterios Antoniou, Dimitris Tatsis, Alexandros Louizakis, Kalliopi Domvri, Konstantinos Paraskevopoulos

**Affiliations:** 1 Oral and Maxillofacial Surgery Department, Aristotle University of Thessaloniki, Thessaloniki, GRC; 2 Histology-Embryology Department, Aristotle University of Thessaloniki, Thessaloniki, GRC

**Keywords:** craniotomy, heterotopic ossification, myositis ossificans, temporalis muscle, trismus

## Abstract

Myositis ossificans (MO) is a rare benign condition characterized by heterotopic bone formation within soft tissues, with masticatory muscle involvement being exceptionally uncommon. This case report describes a 45-year-old male patient who developed progressive trismus and a preauricular mass six months after right frontotemporal craniotomy for meningioma resection. Imaging revealed an ossified lesion in the temporalis muscle, consistent with post-traumatic MO. Surgical management involved zygomatic arch osteotomy and coronoidectomy, restoring intraoperative mouth opening to 23 mm. Histopathology confirmed mature lamellar bone, supporting the diagnosis. At six-month follow-up, the patient achieved full functional recovery with no recurrence. This case highlights the importance of considering MO in post-craniotomy trismus and the role of timely surgical intervention in established lesions.

## Introduction

Myositis ossificans (MO) is a rare benign pathology characterized by unusual heterotopic bone formation within soft tissues, most commonly affecting skeletal muscle. MO usually occurs in extremities affecting large skeletal muscles; it is exceptionally rare in the head and neck region and poses a distinctive diagnostic challenge. MO is generally classified into two forms: myositis ossificans ambiguous progressiva (fibrodysplasia ossificans progressiva (FOP)), a genetic and systemic condition with diffuse ossification and congenital disorders, and myositis ossificans traumatica (MOT), a localized form that occurs after trauma, surgical intervention, or repeated mechanical stress [1-4].

In the maxillofacial region, MO usually occurs in the masticatory muscles, such as the masseter, temporalis, and medial pterygoid [5-7]. The majority of cases are often associated with prior trauma, including dental extractions, local anesthetic injections, mandibular fractures, or surgical access for craniofacial procedures [3,8-10]. The common clinical findings are progressive trismus, localized swelling, and a palpable solid mass. Due to the non-distinct symptoms, appropriate management delays as MO is frequently misdiagnosed as a space-occupying lesion, temporomandibular joint (TMJ) ankylosis, or malignant tumor [5,11,12]. MO involving the temporalis muscle is extremely rare, especially when occurring as a complication of neurosurgical procedures. Frontotemporal craniotomies often require dissection and elevation of the temporalis muscle, which might lead to minor but clinically significant muscle trauma capable of promoting heterotopic ossification. However, only one case has been reported in the current literature associating post-craniotomy changes with the development of temporalis MO, highlighting this rare entity [11].

Radiological findings play a crucial role in making the right diagnosis. Computed tomography (CT) is the gold standard as far as the imaging modalities are concerned, showing the classic zonal ossification pattern characterized by a central radiolucent or less ossified area and a peripheral mature bone [2,10,13,14]. Magnetic resonance imaging (MRI) may offer some useful information in the early inflammatory phase, but it lacks specificity [14,15]. Histologically, MO is characterized by a distinct zonal architecture with an inner zone of fibroblastic proliferation, an intermediate zone of osteoid formation, and an outer border of mature lamellar bone [14,16,17]. This microscopic pattern helps in distinguishing MO from other malignant lesions, such as osteosarcoma or chondrosarcoma, which lack this cellular architecture and present atypia [18].

Limitation of function and lesion maturity determine the right management in most cases. Conservative therapy can be sufficient for asymptomatic or early-stage cases; however, surgical intervention is the treatment of choice in patients with functional impairment or pain [2]. Timing of surgery is crucial; early excision risks recurrence, while delayed intervention until ossification maturity improves outcomes [14,15]. Postoperative rehabilitation, including physiotherapy, is essential for preserving the range of motion and preventing fibrosis [15].

Temporalis muscle-related trismus following neurosurgical approaches is significant due to its functional implications in mastication and mouth opening. Delayed recognition may lead to prolonged trismus, patient discomfort, and diminished quality of life. The aim of this article is to present a rare case of temporalis muscle MO following neurosurgical intervention and to review the current literature on MO in the head and neck region.

## Case presentation

A 45-year-old male patient was referred to the outpatient clinic of our department with progressive limitation of mouth opening, after a right frontotemporal craniotomy for resection of a meningioma six months prior. The patient also reported a decreased nutritional intake by mouth and weight loss due to a restricted soft diet, and impaired speech. There was no history of temporomandibular joint dysfunction or local trauma in the area postoperatively. The only ongoing medication was levetiracetam, administered twice daily after the neurosurgical procedure, with no other coexisting comorbidities. Clinical examination revealed pronounced trismus with a maximum interincisal opening of 5 mm. Bilateral lateral excursion of the mandible was severely restricted. A firm, immobile, preauricular mass was palpable over the mid-portion of the right zygomatic arch. Cranial nerve examination was otherwise unremarkable.

**Figure 1:**
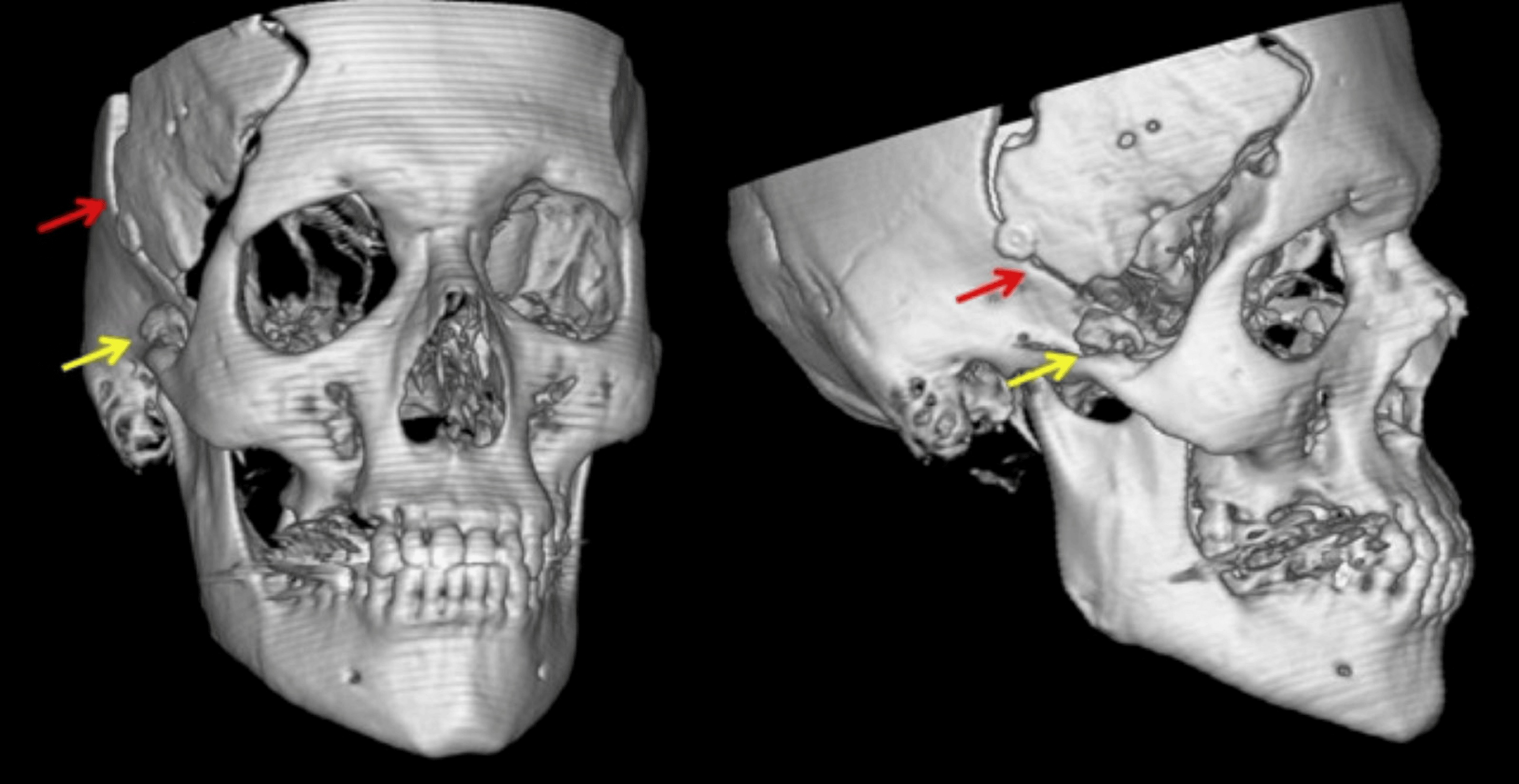
3D reconstruction of the CT scan of the patient The ectopic ossified lesion in the temporal fossa can be distinguished anteromedial to the right zygomatic arch (yellow arrow). The previous right frontotemporal craniotomy site can also be seen (red arrow). CT: computed tomography

Thin-slice (1.25 mm) non-contrast CT of the facial bones revealed a hyperdense, irregular, ossified lesion (length: 1.82 cm, width: 1.35 cm, height: 1.32 cm) medial to the right zygomatic arch, anterior to the coronoid process. The measured Hounsfield unit (HU) for this lesion was 600, indicative of mature bone (Figure 1). The lesion appeared to originate from the temporalis muscle and extended to attach to the zygomatic arch, causing a mechanical impediment to the anterior movement of the mandibular condyle. The combination of radiological and clinical findings indicated myositis ossificans of the temporalis muscle as a diagnosis due to secondary surgical trauma during craniotomy.

**Figure 2:**
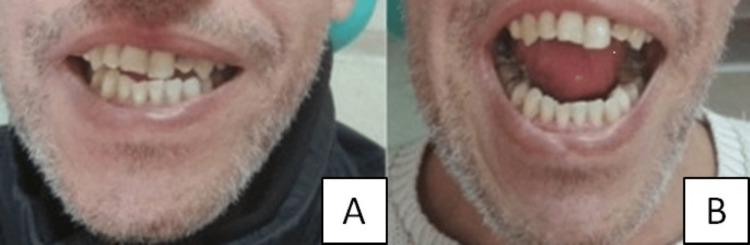
(A) Preoperative maximum interincisal opening and (B) postoperative maximum interincisal opening

Informed consent of the patient for treatment and publication has been obtained prior to surgery. The patient was scheduled for surgical intervention with the goal of re-establishing functional mandibular mobility. Due to the severity of trismus, fiber-optic nasotracheal intubation was performed under general anesthesia. Surgical access was obtained via the existing right preauricular incision from the prior craniotomy. A double osteotomy of the zygomatic arch both anterior and posterior to the lesion was performed to allow en bloc resection of the ossified mass along with the involved segment of the zygomatic bone. Furthermore, a coronoidectomy was performed through a vestibular incision to release the temporalis muscle to the coronoid process. Intraoperative assessment revealed immediate improvement, with maximum interincisal opening (MIO) increasing from 5 mm to 23 mm and improved lateral excursion of the mandible from 1 mm to 6 mm (Figure 2). The patient had an uneventful postoperative course and was discharged three days after surgery. The MIO reached a normal range after six months with passive and active physiotherapy.

**Figure 3:**
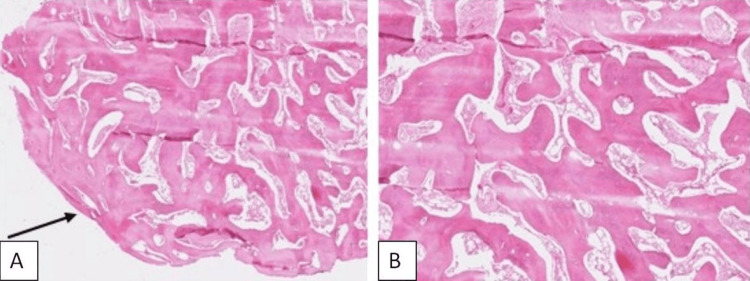
Pathology report: (A) peripheral mature lamellar bone (black arrow) (hematoxylin and eosin, ×20) and (B) peripheral mature lamellar bone (hematoxylin and eosin, ×40)

**Figure 4:**
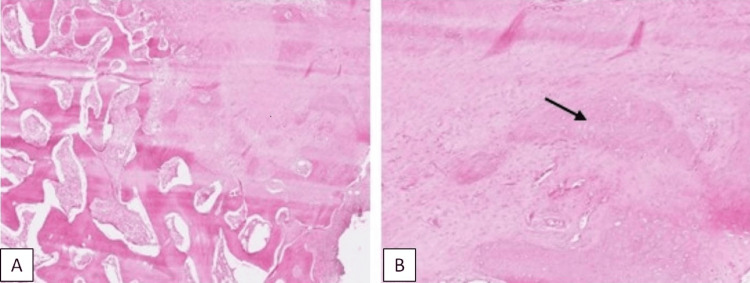
Pathology report: (A) transition zone from immature to mature bone tissue (hematoxylin and eosin, ×20) and (B) immature bone (black arrow) with fibroblasts and vascularity (hematoxylin and eosin, ×40)

The pathology report of the excised specimen revealed the presence of mature lamellar bone consistent with myositis ossificans (Figures 3, 4).

## Discussion

Myositis ossificans (MO) is a rare but notable pathology in the head and neck region, especially when affecting the masticatory muscles. Our case of a 45-year-old male patient with gradually deteriorating mouth opening following craniotomy in the frontotemporal area adds to the limited reported cases with temporalis muscle involvement, a muscle less frequently affected compared to the masseter or medial pterygoid. Despite MO being an uncommon entity in the head and neck region, understanding the clinical and radiological findings and the therapeutic options for MO in this region is essential for appropriate management.

Two types of MO have been reported: myositis ossificans traumatica, a localized acquired form, and fibrodysplasia ossificans progressiva, a rare autosomal dominant condition with systemic progression [2,5,6]. Most of the cases in the craniofacial area are classified as MOT with distinct predisposing factors such as mandibular fractures, dental procedures, injections of local anesthetics, orthognathic surgery, or radiation [14-16]. The pathophysiology behind MOT remains incompletely understood, but surgical trauma appears to trigger an abnormal healing response, characterized by fibroblastic proliferation, osteoid formation, and eventual ossification. Inflammatory mediators, local hematoma formation, and upregulation of bone morphogenetic proteins (BMPs), particularly BMP-2 and BMP-4, are believed to play central roles in promoting mesenchymal cell differentiation into the osteogenic lineage. Genetic predisposition, although primarily involved in FOP, may also act as a cofactor in some localized cases, affecting individual susceptibility to heterotopic ossification following trauma [18].

In our case, although no direct trauma to the masticatory muscles of the right area was reported, the dissection of the temporalis muscle during craniotomy appears to have triggered heterotopic bone formation due to indirect trauma. This is consistent with reports by Aoki et al. [4] and Nemoto et al. [16], who reported MO of the temporalis and lateral pterygoid muscles after mandibular surgery. Similar trigger mechanisms are indicated in cases where MO followed minor or subclinical trauma, such as repeated dental injections [1-3]. The characteristic clinical finding of MO in the head and neck region is progressive trismus, particularly when affecting the masticatory muscles. In some cases, a solid, painless mass can also be present [9,10]. In our case, the patient presented with gradual deterioration of mouth opening along with the emergence of a firm mass at the zygomatic arch area without any reports of further trauma after the neurosurgical procedure, and also no underlying systemic disease or medications known to cause ectopic bone formation or connected to the pathogenesis of MO.

Radiological findings play a crucial role in the differential diagnosis of MO. Computed tomography is the gold standard due to its ability to detect early ossification in the affected muscles and define its anatomical extent [13-15]. The characteristic “zonal” ossification pattern with peripheral mature bone and a central radiolucent core helps to distinguish MO from malignancies such as osteosarcoma or chondrosarcoma, which exhibit irregular mineralization and cortical bone erosion [17]. In our patient, CT scan revealed a densely calcified lesion medial and superior to the zygomatic arch originating from the temporalis muscle, restricting the movement of the coronoid process, which is consistent with similar reports in the literature [10,15,17]. MRI, on the other hand, can be used in early stages when ossification is not evident, but it can be misleading because it lacks specificity [2,3]. In the early stages, MO may appear as an edematous soft tissue mass, which can radiologically mimic a malignant neoplasm. For this reason, radiological features should be analyzed alongside clinical findings and, when indicated, with the pathology report after biopsy. The classic microscopic findings include a triphasic zonal pattern with a central area of immature fibroblasts, an intermediate zone with osteoid and cartilage formation, and a peripheral zone of mature lamellar bone. This layered architecture distinguishes MO from sarcomas, which lack such zonation and display atypia, mitoses, and permeative growth [1].

Treatment management depends on the stage of MO and the severity of clinical symptoms. In the acute phase, conservative treatment is preferred, especially if functional impairment is minimal. However, in cases with established ossification and significant limitation of mandibular mobility, surgical intervention is required [14,15]. The timing of surgery is crucial because early excision of the lesion during the inflammatory or immature ossification phase can lead to recurrence. Most of the literature suggests delaying surgical intervention until the mature lesion is radiographically evident (usually after six months), thereby reducing the risk of recurrence [2,4,14]. In our case, surgical intervention was implemented due to severe trismus and radiological evidence of mature ossification. The patient underwent zygomatic arch osteotomy and coronoidectomy to access and remove the lesion. The same surgical management has been employed successfully in previous reports for MO involving the temporalis muscle to alleviate the mechanical restriction on mandibular movement [14,19]. Intraoperatively, a well-circumscribed ossified solid mass was identified and resected en bloc with the coronoid process. The postoperative course was favorable, with a distinct increase in maximum interincisal opening from 5 mm prior to surgery to 23 mm immediately afterward. This highlights the value of a carefully planned surgical approach in cases where conservative management proves insufficient. A well-defined physiotherapy and rehabilitation protocol is crucial in minimizing the risk of postoperative fibrosis and recurrence and achieving a favorable long-term surgical outcome. In our case, the patient followed a postoperative physiotherapy regimen with passive and active mouth opening exercises. At nine-day follow-up, improvement in mouth opening was notable, which is consistent with other reports where early mobilization postoperatively provides improved outcomes and minimized relapse [2,14,15].

Our case is in accordance with the growing recognition of iatrogenic MO as a postoperative complication, especially in neurosurgical and maxillofacial procedures. Aoki et al. [4] and Nemoto et al. [16] reported MO in the temporalis and lateral pterygoid muscles, respectively, following open reduction of mandibular fractures, highlighting the probability for MO to occur in any masticatory muscle after trauma. Similarly, Karaali et al. [20] described MO in the medial pterygoid muscle after a molar extraction, further highlighting that even minor trauma may be capable of triggering MO [4,16]. Differentiating MO from other causes of trismus is crucial. Temporomandibular joint ankylosis, masticatory space infections, fibrous dysplasia, and ossifying fibromas must be considered in the differential diagnosis. MO may be misdiagnosed as TMJ ankylosis due to similar clinical features. However, computed tomography facilitates distinction between the two conditions, with MO presenting as an extra-articular lesion and characterized by distinctive ossification patterns [15]. The rarity of temporalis muscle involvement, as reported in our case, highlights the importance of clinical suspicion in patients after craniofacial or neurosurgical procedures with delayed onset of trismus. Early identification through imaging and multidisciplinary evaluation can facilitate timely intervention and functional recovery. While recurrence is uncommon after complete resection of mature lesions, long-term follow-up is recommended to monitor for regrowth and maintain mandibular mobility [5,14,15].

## Conclusions

This case illustrates a rare presentation of myositis ossificans involving the temporalis muscle after craniotomy, probably triggered by indirect surgical trauma. Unlike previously reported cases, there was no direct injury to the muscle, highlighting a broader mechanism of heterotopic ossification in the craniofacial region. This rare entity should always be considered by clinicians in the differential diagnosis of patients with persistent trismus following neurosurgical procedures, without common etiologic factors. To improve early detection, routine postoperative assessment of mandibular mobility should be considered, with prompt referral to oral and maxillofacial surgeons when functional limitation is observed. Early implementation of CT imaging is crucial for the accurate diagnosis of MO and optimal surgical planning. Surgeons performing craniotomies should aim to minimize temporalis muscle dissection where possible to reduce the risk of ectopic ossification. Greater awareness of this uncommon complication may lead to earlier multidisciplinary intervention and improved patient outcomes.
